# ‘I cannot see ahead’: psychological distress, doomscrolling and dark future among adult survivors following M_w_ 7.7. and 7.6 earthquakes in Türkiye

**DOI:** 10.1186/s12889-023-17460-3

**Published:** 2023-12-15

**Authors:** Aslı Kartol, Servet Üztemur, Pınar Yaşar

**Affiliations:** 1 Department of Psychological Counseling and Guidance, Nizip Faculty of Education, Gaziantep University, Gaziantep, Turkey; 2 Department of Social Studies Education, Faculty of Education, Anadolu University, Eskişehir, Turkey; 3 Department of Psychological Counseling, Guidance Gaziantep University, Gaziantep, Turkey

**Keywords:** Doomscrolling, Depression, Anxiety, Stress, Earthquake, Dark future

## Abstract

**Background:**

The earthquakes, which occurred on 6 February 2023, affecting a total of eleven provinces in Türkiye, with magnitudes of 7.7 and 7.6, and killing around 50,000 people, caused the greatest loss of life compared to previous earthquakes in Türkiye. In this study, we analyse the psychological status of the adult individuals who experienced the earthquakes three months after the earthquakes in terms of different variables.

**Methods:**

In this research, an analytical cross-sectional study was conducted by applying face-to-face and online questionnaires to 402 adult individuals who had experienced the earthquake. The Depression, Anxiety and Stress Scale (DASS-21), the Dark Future Scale and Doomscrolling Scale were applied. The relationships between variables are discussed in this study using the predictive correlational model.

**Results:**

The results revealed that the participants had very high levels of future anxiety and moderate levels of doomscrolling after the earthquake. Although their psychological distress levels were relatively low, as the psychological distress levels of adult individuals who experienced the earthquake increased, their doomscrolling also increased. In addition, as individuals’ future anxiety increases, their doomscrolling increases similarly. The mediation analysis reveals that the mediating role of future anxiety between psychological distress and doomscrolling is significant (*p* < .001).

**Conclusion:**

Individuals are more exposed to negative news streams in negative life events that occur beyond their control and they need to search for information. Increased levels of depression, anxiety and stress lead to more exposure to this flow. In addition, future anxiety is also an important trigger of this behaviour. The effects of psychological distress on individuals who survived the earthquake are discussed in the context of the literature.

## Background

On February 6, 2023, two consecutive earthquakes of magnitudes 7.7 and 7.6 occurred in Türkiye and also occurred simultaneously in Syria, affecting eleven different provinces and killing 50,783 people in Türkiye according to the official data [1]. As a result of these earthquakes, many people became homeless, resettled in relatives’ houses or started to live in container cities. According to the official authorities, more than 48,000 people lost their lives. According to estimates, more than 500,000 houses were destroyed. Many people, especially those living in Kahramanmaraş and Hatay, were left homeless. Tents and container cities have been set up in the region. Most of the disaster victims had to migrate to different parts of Türkiye. The total population of the 11 provinces affected by the earthquake was around 14 million in 2022. It was estimated that 3 million people migrated to different parts of Türkiye in the first month following the earthquake. Official reports put the estimated material damage of the earthquake at 103.6 billion dollars, corresponding to approximately 9% of Türkiye’s national income in 2023 [2]. Since earthquakes are natural disasters that cannot be predicted or controlled and they have life-threatening effects and cause large-scale destruction [3], problems that negatively affect mental health may occur in individuals afterwards [4]. Studies conducted after major disasters reveal that the mental health of individuals—who survived disasters such as war [5], landslides [6], floods [7], and tsunamis [8] is negatively affected.

Significant losses, such as homelessness and the loss of relatives after an earthquake create many psychological problems [3]. Depression and posttraumatic stress disorder (PTSD) are frequently seen together after traumatic events [5, 9]. When the studies conducted after an earthquake are examined, many studies show that post-traumatic stress disorder, depression, anxiety and stress symptoms occur as a result [10–15]. At the same time, studies report that individuals have intense anxiety symptoms after an earthquake [16, 17]. Longitudinal studies show that depression and anxiety symptoms persist even years later [11, 18–21]. Since depression, anxiety and stress symptoms continue for a long time after an earthquake, more research on this topic is extremely important for mental health theories.

Individuals tend to spend more time on social media and seek information from news sources during negative life events [22]. Emerging in 2020 with the pandemic, Doomscrolling, also known as doomsurfing, is characterised by uncontrollably and compulsively continuing to search and scroll current negative news even if it creates unhappiness and despair [23]. More specifically, individuals spend a lot of time reading bad news on the phone or computer after negative life events even though it is known to cause negative feelings in them [24]. It is a human impulse to stay in the flow of information due to the bad feeling created by uncertainty [25] because people need to seek information to adapt to changing life conditions [26]. Studies have shown that doomscrolling potentiates psychological distress, depressive and anxious affect, and PTSD symptoms [27–29].

After an earthquake, individuals often experience loss of life, property or work. In the process of continuing their lives with all these losses, they may be occupied with automatic thoughts about the possibility of another earthquake. This situation creates physical health problems, adaptation problems, security problems due to looting, and many negative situations. Individuals may experience future anxiety due to fear of dangerous situations that might happen in the future [30]. Satıcı et al. [31] revealed that fear of earthquakes increases psychological distress and reduces harmony in life and mental well-being. In a global context, individuals who experience dangerous life events and think about the possibility of relieving themself tend to experience future anxiety [30]. Also, Bırni et al. [32] discussed the effects of the February 6, 2023 earthquake in Türkiye on mental health. Studies conducted during the COVID-19 pandemic period indicate that people experience future anxiety in the face of dangerous and negative life events [33–35]. Because earthquakes are a disaster whose impact is unpredictable and remains uncertain, it is normal for individuals to feel anxiety. We, mental health experts, know that anxiety is a protective emotion in times of danger. Still, since uncontrolled anxiety negatively affects mental health, it is possible to control anxiety with some interventions.

### Present study

How people cope with uncertainty has been a common research topic for many years [36]. When individuals face difficult situations, they search for information as a coping mechanism. This search is protective against possible future threats [26]. Previous research shows that individuals who exhibit excessive doomscrolling after traumatic events experience high levels of depression, anxiety, and stress [37, 38]. Doomscrolling may lead individuals to see the future more pessimistically and experience hopelessness [39]. High rates of depression, anxiety and stress after traumatic experiences may be associated with future anxiety [40]. This study is critical to the theory in terms of addressing the concept of doomscrolling, which has entered the literature during the pandemic, and the concept of future anxiety, which has not been the subject of a great deal of research. It is a possible expectation that individuals will be psychologically affected by this process after an earthquake disaster and the effects will continue afterwards [41]. In this context, our study aims to examine the mediating role of future anxiety in the relationship between psychological distress (depression, anxiety and stress) and doomscrolling levels of adults during the recent earthquakes. This study may raise awareness regarding the importance of understanding the mental health problems of individuals after earthquake trauma, the anxiety they feel about the future and the importance of controlling the news flow. Şalcıoğlu et al. [42] emphasize in their post-earthquake study that earthquakes have long-term psychological consequences, especially for earthquake victims exposed to high levels of trauma and long-term mental health service policies are needed for earthquake victims. In this respect, the results of this study will contribute to the intervention programmes to be created by mental health specialists and serve as a guide.

**Fig. 1:**
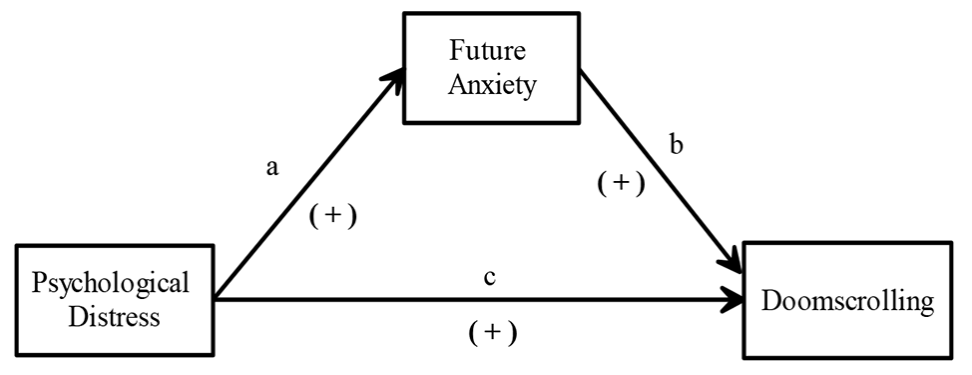
Relationships between psychological distress, future anxiety, and doomscrolling

We propose that psychological distress has an indirect effect on doomscrolling through future anxiety. Based on the assumption that future anxiety has a mediating effect on the relationship between psychological distress and doomscrolling, a model revealing the relationships between these three variables was created based on the relevant literature and is shown in Fig. 1.

The following research question was answered, and the hypotheses given below were tested:

Research Question: What is the level of psychological distress, future anxiety and doomscrolling of the participants and how, and in what direction, are there relationships between these three variables?

- Hypothesis 1 (H1): Psychological distress predicts doomscrolling positively (path c).
- Hypothesis 2 (H2): Psychological distress predicts future anxiety positively (path a).
- Hypothesis 3 (H3): Future anxiety predicts doomscrolling positively (path b).
- Hypothesis 4 (H4): In the model tested, future anxiety has a mediating effect between the predictor (psychological distress), and the predicted (doomscrolling).

## Method

### Research design

In this study, a multifactorial correlational design was used to reveal the structural relationships between the psychological distress, future anxiety and doomscrolling of adult individuals who experienced the earthquakes of February 6th. In predictive correlation studies, the relationships between variables are examined and an attempt is made to predict the other based on one of the variables. Multifactorial predictive correlational designs can be intended to test only direct relationships or both direct and indirect relationships [43]. In this study, we identified psychological distress as a predictor, future anxiety as a mediator and doomscrolling as a predicted variable.

### Participants, procedure and ethics

The study was conducted with 402 adults who had personally experienced the earthquakes that occurred in southern Türkiye on February 6, 2023. Since the psychological effects of the earthquake continued during the data collection phase, the participants were randomly selected by convenience sampling method. The data were administered both face-to-face and online via Google Forms with volunteer participants between April 1 and May 15, 2023. The study procedures were designed in line with the Declaration of Helsinki guidelines. Informed consent was obtained from all participants. All participants voluntarily participated in the study and no payment was made to them. Participants were assured that their personal information would be kept confidential. After the data collection process, they were reminded that they could opt out of the study’s participants if they wished. The approval of the Gaziantep University Social Sciences Ethics Committee was obtained before the data collection process (Ethics Number: 329,482).

### Data collection

*Depression, Anxiety, and Stress Scale*: The DASS21 Scale [44, 45] was used to assess psychological distress levels. The scale is a four-point Likert-type scale ranging from 0 (not at all favourable to me) to 4 (completely favourable to me). There are twenty-one items on the scale, which consists of three sub-dimensions; depression, anxiety and stress (Example item: ‘I realised that I could not experience any positive emotions’). Higher scores indicate more psychological distress. The fit indices of the translated scale are excellent: χ2/df: 2.842, GFI: 0.99, AGFI: ,98, RMR: 0.05, NFI: 0.98, RMSEA: 0.04 [43]. Confirmatory factor analysis (CFA) was conducted to ensure construct validity. The results confirm the original structure of the scale and the fit indices are acceptable: χ2/df = 2.888; IFI = 0.94; RMSEA = 0.06; SRMR = 0.03; CFI = 0.91; and TLI = 0.93 [46]. The internal consistency coefficient (Cronbach’s Alpha) of the original scale for anxiety, depression, and stress subscales, respectively, were as follows: 0.81, 0.82, 0.76. In this study, the internal consistency coefficient (Cronbach Alpha) for the overall scale was 0.96.

*Doomscrolling Scale*: The Doomscrolling Scale [29, 47] was used to assess doomscrolling. The scale is a 7-point Likert scale ranging from 1 (strongly disagree) to 7 (strongly agree). There are fifteen items in the scale consisting of a single dimension (Sample item: ‘I feel like I am addicted to negative news’). Higher scores indicate more doomscrolling. The fit indices of the translated scale are acceptable: χ(89, N = 378) = 402.57, *p* < .001; CFI = 0.92, NFI = 0.90, IFI = 0.92, SRMR = 0.04. To ensure construct validity, CFA results confirmed the original structure of the scale and the fit indices were acceptable: χ2/df = 3.281, IFI = 0.97, RMSEA = 0.07, SRMR = 0.02, CFI = 0.97, and TLI = 0.96. In the original scale, the internal consistency coefficient (Cronbach Alpha) for the overall scale was 0.94. In this study, the internal consistency coefficient (Cronbach Alpha) for the overall scale was 0.96.

*Dark Future Scale (DFS)*: The Dark Future Scale [30, 48] was used to assess future anxiety. The scale is a 6-point Likert scale ranging from 1 (strongly disagree) to 6 (strongly agree). It consists of five items in a single dimension (Example item: ‘Sometimes the thought of facing life’s crises or difficulties scares me a lot’). Higher scores indicate more future anxiety. The fit indices of the translated scale are excellent: χ2/df = 2.194, TLI = 0.96, CFI = 0.98, RMSEA = 0.07, and SRMR = 0.02. To ensure construct validity, CFA results confirmed the original structure of the scale and the fit indices were acceptable: χ2/df = 3,694, IFI = 0.99, RMSEA = 0.08, SRMR = 0.01, CFI = 0.99, and TLI = 0.98. In the original scale, the internal consistency coefficient (Cronbach Alpha) for the overall scale was 0.79. In this study, the internal consistency coefficient (Cronbach Alpha) for the overall scale was 0.92.

### Data analysis

In the first stage, we used descriptive statistics. In the second stage, we calculated Pearson product-moment correlation coefficients to determine the relationships between the variables. In the third stage, we used hierarchical regression analysis to reveal the mediating effect of future anxiety in the relationship between psychological distress and doomscrolling. To check for multicollinearity, we checked whether the tolerance values were greater than 0.20 and whether the variance inflation factor (VIF) was less than 10 [49]. To test the statistical significance of the mediating effect of future anxiety, we applied the bias-corrected bootstrapping method proposed by Hayes [50] with the “SPSS Process Macro” plug-in. We increased the number of samples to 5,000 by the random sampling method to create 95% confidence intervals. The absence of a zero value between confidence intervals indicates that the mediating effect tested in the model is statistically significant [50]. SPSS version 25 was used in the analyses. We accepted 0.05 for the significance value.

### Findings

Information about the participants is given in Table 1.

**Table 1:** Participant information (*N* = 402)

Variable	Category	*N*	Percentage %
**Gender**	Female	310	77,1
	Male	92	22,9
	**Total**	**402**	**100**
**Age**	18–30	208	51,7
	31–40	122	30,3
	41–50	57	14,2
	51 and above	15	3,7
	**Total**	**402**	**100**
**Education Level**	Primary School	18	4,5
	Middle School	19	4,7
	High School	44	10,9
	Bachelor’s degree	284	70,6
	Postgraduate	37	9,2
	**Total**	**402**	**100**
**Marital Status**	Married	184	45,8
	Single	218	54,2
	**Total**	**402**	**100**
**Loss of family members due to disaster**	Yes	32	8
	No	370	92
	**Total**	**402**	**100**
**Loss of relatives or friends due to disaster**	Yes	206	51,2
	No	196	48,8
	**Total**	**402**	**100**
**Current place of residence**	Tent/Container	84	20,9
	Own House	286	71,1
	Home of relatives	32	8
	**Total**	**402**	**100**
**Being trapped under rubble during an earthquake**	Yes	14	3,5
	No	388	96,5
	**Total**	**402**	**100**
**Damage to house**	Heavy damage	41	10,2
	Little damage	143	35,6
	No Damage	161	40,0
	Moderate damage	21	5,2
	Destroyed	36	9,0
	**Total**	**402**	**100**

As can be seen in Table 1, more than 50% of the participants are between the ages of 18–30, which can be considered in the young adult category. 70.2% of the participants are university graduates. The rate of married participants is around 46%. 8% of the participants lost a family member in the earthquake. The proportion of the participants whose relatives died due to the earthquake is 51.2%. Approximately 25 per cent of the participants’ houses were destroyed or became uninhabitable. The rate of the participants who are still living in tents or containers is around 20 per cent. More than 70 per cent of the participants are women.

## Descriptive statistics and correlations

Descriptive statistics and correlation values related to psychological distress, future anxiety and doomscrolling are shown in Table 2.

**Table 2:** Descriptive statistics and correlation analysis (*N* = 402)

Variable	Mean	S. Dev.	Skewness	Kurtosis	(1)	(2)	(3)
1. Doomscrolling	2.70	1.68	0.956	− 0.046	1	0.536^**^	0.470^**^
2. Future Anxiety	4.03	1.48	− 0.367	− 0.876		-	0.649^**^
3. Psychological distress	1.44	0.81	0.048	− 0.915			-

**p < .01

As can be seen in Table 2, there is a moderate positive correlation between doomscrolling and future anxiety and psychological distress. There is a moderate positive significant correlation between future anxiety and psychological distress. Considering the skewness and kurtosis values, it can be said that the data set is normally distributed (kurtosis and skewness ≤ |1|) [46].

### Mediation analyses

To test the mediating effect of future anxiety, we conducted a three-stage hierarchical regression analysis. In the first stage, the dependent variable (doomscrolling) was predicted by the independent variable (psychological distress). In the second stage, the mediator variable (future anxiety) was predicted by the independent variable. In the third stage, the dependent variable was predicted by both the predictor variable and the mediator variable. Gender and marital status were included in all the analyses as control variables. The results of the three-stage hierarchical regression analysis are given in Table 3.

**Table 3:** Hierarchical regression analysis predicting doomscrolling

Regression Equation	Dependent Variable	Independent Variable	B	β	t	r^2^	Δr^2^	ΔF
1	Doomscrolling	Gender	− 0.189	− 0.056	-1.2657	0.224	-	38.3542
		Marital status	0.030	0.009	0.2068			
		Psychological distress	0.980	0.473	10.7037^***^			
2	Future Anxiety	Gender	0.056	0.018	0.4945	0.425	-	98.0341
		Marital status	0.178	0.059	1.5669			
		Psychological distress	1.18	0.646	16.9755^***^			
3	Doomscrolling	Gender	− 0.214	− 0.063	-1.5296	0.317	0.09	46.1502
		Marital status	− 0.050	− 0.014	− 0.3567			
		Psychological distress	0.441	0.213	3.9098^***^			
		Future Anxiety	0.454	0.402	7.3598^***^			

**Fig. 2:**
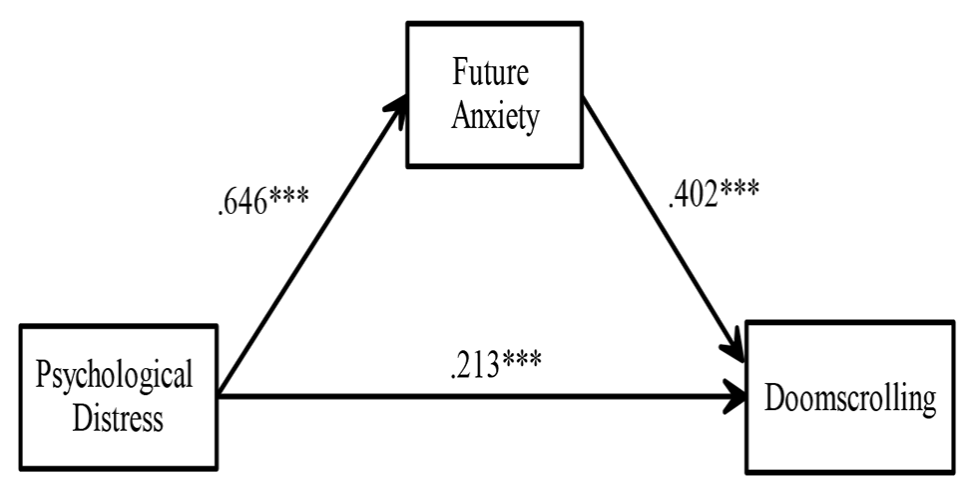
Standardized beta coefficients for the mediating effect of future anxiety on the relationship between psychological distress and doomscrolling, *N* = 402, ^***^= *p* < .001

***= *p* < .001,

As can be seen in Table 3, in the first stage (Regression Eq. 1), a direct path was established between psychological distress and doomscrolling (H1), and the independent variable predicted the dependent variable significantly and positively (β = 0.47, t = 10.7037, *p* < .001). According to these findings, H1 was confirmed and 22.4% of the variance in doomscrolling was explained by psychological distress. In the second stage (Regression Eq. 2), psychological distress positively predicted future anxiety and H2 was accepted (β = 0.64, t = 16.9755, *p* < .001). Accordingly, 42.5% of the variance in future anxiety is explained by psychological distress. In the third stage (Regression Eq. 3), after the mediating variable was included in the model, although psychological distress predicted doomscrolling significantly, the effect coefficient of psychological distress decreased (β = 0.21, t = 3.9098, *p* < .001). Future anxiety predicted doomscrolling positively, and this effect was statistically significant and H3 was confirmed (β = 0.40, t = 7.3270, *p* < .001). According to these findings, it can be said that future anxiety mediates the relationship between psychological distress and doomscrolling. The mediating variable analysis is visualised in Fig. 2.

According to Fig. 2, the indirect effect of psychological distress on doomscrolling through future anxiety (0.64 × 0.40 = 0.26) corresponds to 55% (0.26 / 0.47 = 0.55) of the total effect (0.26 + 0.21 = 0.47). With the addition of the mediator variable to the model, an increase of 0.09 in the variance explained in the dependent variable occurred. In addition, 31.7% of the variance in the dependent variable is explained by the independent variable, mediator variable and control variable together. When the mediator variable is not included in the model, this rate decreases to 22.4%. Control variables (gender, and marital status) have no significant effect on the dependent variable and mediator variables.

To test the statistical significance of the mediating effect of future anxiety, the ‘bias-corrected bootstrapping’ method was applied with an ‘SPSS Process Macro’ plug-in as suggested by Hayes [50]. The indirect effect coefficients and 95% confidence intervals are given in Table 4.

**Table 4:** The bootstrapping for the partial mediation model (*N* = 402)

			95% Confidence Interval	
**Model Paths**	**SPC**	**SE**	**Lower**	**Upper**
DASS→FA→DOOM	0.26^**^	0.03	0.201	0.320

DASS = Psychological distress, FA = Future Anxiety, DOOM: Doomscrolling, SPC = Standardised Path Coefficient, SE. = Standard Error, ^**^p < .01,

As can be seen in Table 4, the indirect path coefficient of future anxiety is significant after 5,000 bootstrap procedures, and there is no zero between the confidence intervals (bootstrap coefficient = 0.26, 95% Confidence Interval = 0.20, 0.32). According to these findings, H4, which suggests that future anxiety will mediate the relationship between psychological distress and doomscrolling, was confirmed.

## Discussion

In this study, the effects of psychological distress and future anxiety levels on doomscrolling are examined. The first finding of the study is that psychological distress positively predicts doomscrolling (supporting H1). Similar to this finding, a study conducted by Satici et al. [47], which shows that there is a positive relationship between psychological distress and doomscrolling in the literature, supports our finding. The first studies on the concept of doomscrolling, which entered our lives with the pandemic, were carried out during COVID-19. Therefore, there are studies investigating the relationship of this concept with other mental health variables during the COVID-19 process [23]. Earthquakes are negative life events that cause great material and moral damage and remain uncertain, as does the COVID-19 process. Therefore, similar psychological outcomes are expected. Anand et al. [37], on the other hand, state that individuals’ tendency to spend more time on the Internet for work or leisure activities increased during the isolation process in their study conducted during the COVID-19 process and their study revealed that doomscrolling predicts psychological distress. Karakose et al. [51] found a positive relationship between psychological distress and social media addiction in their study conducted during the COVID-19 process. Chen et al. [52] reveal that psychological distress levels increased as Internet-related behaviour increased during the COVID-19 process. There are also new studies revealing significant relationships between doomscrolling and depression in climate change [53, 54]. Individuals seek information to make sense of a situation, especially in situations they cannot control, to reduce their anxiety and fears [37]. In negative life events (natural disasters, epidemics, and so on) especially, and situations affecting society in general (e.g., elections), individuals feel the need to obtain more information and they may fall into a deadlock as they encounter negative news from the digital media. This vicious cycle of negative news triggers negative emotions in individuals and causes them to experience a negative emotional state. Therefore, it is thought that preventive intervention studies that improve an individual’s ability to avoid negative news will lead to a reduction in psychological distress and an increase in well-being.

The second result of the study is that psychological distress significantly predicts future anxiety in a positive direction (supporting H2). Dey et al. [40] found that individuals with high levels of depression anxiety stress also had high levels of future anxiety. This result is in line with our results. Miranda and Mennin [55] emphasized in their study that individuals who were depressed were more likely to negatively evaluate the future and to think that positive events are not to happen. In summary, individuals with high levels of depression and general anxiety disorder tend to develop a negative perspective towards the future [56]. Such negative emotions come to the fore in these disorders and negatively affect the mental health of individuals. In this respect, individual or group psychological counselling programmes aimed at reducing psychological distress will help to reduce future anxiety.

The third finding of the study was that future anxiety positively predicted doomscrolling (supporting H3). In their study, Shabahang et al. [39] found that future anxiety increased as the level of doomscrolling increased and emphasised that doomscrolling is a risky activity that has the potential to increase worrisome thoughts about the future and hopelessness. Exposure to negative news flow can increase the level of anxiety by causing individuals to negatively interpret their perspective on life and the future. Therefore, the increase in anxiety levels negatively affects the psychological well-being of individuals. In these respects, intervention studies that can be carried out to reduce future anxiety may have a reducing effect on the commitment of individuals to the doomscrolling behaviour in which they are constantly dragged along in the search for information.

The fourth and final finding of the study is that future anxiety plays a mediating role between psychological distress and doomscrolling (supporting H4). In other words, individuals with psychological distress have more doomscrolling and are more anxious about the future. Many studies conducted during the COVID-19 pandemic reveal that media exposure is closely related to depression and anxiety [57, 58]. Price et al. [28] found that as doomscrolling increased during the COVID-19 process, depression symptoms increased in individuals. A study conducted with university students during the pandemic showed that doomscrolling increased in the face of negative situations [59]. Mobile devices hold the record when it comes to the sum of minutes spent online worldwide, and people can easily connect online anywhere and anytime [60]. In this context, smartphones, and social media news feeds, which are designed to encourage frequent or prolonged interaction, may further increase the need to be informed and facilitate more than usual browsing of negative news [29]. Exposure to negative news streams may cause individuals to be unable to see the future clearly and always experience negative future anxiety. This anxiety process continues as the flow continues and depressive symptoms progress by increasing their severity. As a result of this study, doomscrolling behaviour, which is a new phenomenon, was found to harm mental health. Further studies are therefore needed to identify doomscrolling and its triggers.

### Limitations

There are certain limitations in the present study. First, the findings of the study were based on self-report data which can be subject to various method biases. Second, all the participants were individuals experiencing the earthquakes. This could also be applied to individuals who have not experienced the earthquakes and can be compared. Since people who have not experienced the earthquake are indirectly exposed to the negative effects of the earthquake via social media or television, and since Türkiye is already an earthquake region, it is considered important to conduct these studies on people who have not experienced the earthquake and investigate the level of impact on them. Even when earthquakes occur and end, obsessive thoughts about the earthquake may continue. Third, the sample was skewed towards females, which may have impacted the findings. The other limitation of this study was that data was collected from people affected by the earthquake in Türkiye. By collecting data from individuals in different countries who experienced the earthquake, more accurate generalisations can be made about the mental health effects of the earthquake. Another limitation is that as the individuals in this study experienced the earthquake recently, some of them may still be going through the grieving process. In this case, this may be reflected in their mood when answering the scales. Longitudinal studies can be used to compare the degree of impact of the earthquake over time. Furthermore, the data was collected both face-to-face (this method was preferred, especially for people living in container cities and with internet problems) and via Google Forms online with volunteer participants. Finally, the results were obtained from cross-sectional data, so no causal assumptions can be made.

## Conclusion

In this study, the psychological effects of the earthquake process on individuals were investigated. According to this study, we found that people with high levels of depression, anxiety and stress increased their scrolling behaviour of catastrophic news. We found that individuals with higher levels of psychological distress experienced more anxiety about the future and that this anxiety played a mediating role in doomscrolling behaviour. Unpredictable earthquakes and similar disasters that we cannot control negatively affect the psychology of individuals and their effects continue for a long time. The research has shown that more studies on doomscrolling, which is related to social media use and unconscious technology use, are important for positive mental health. Negative life events have existed and will continue to exist in our lives. In this respect, it is thought that it would be important to benefit from the results of this study while planning the content of preventive, crisis intervention and protective mental health activities in mental health treatment studies. Considering that technology is continually developing and influencing individuals more and more, an increase in studies on the conscious use of technology would positively affect mental health.

## Data Availability

The original form and data of this study are available from the corresponding author upon reasonable request.

## Abbreviations

DASS: 21-The Depression Anxiety and Stress Scale
PTSD: Posttraumatic Stress Disorder
